# Dynamic Neuro-Glial-Vascular Responses in a Mouse Model of Vascular Cognitive Impairment

**DOI:** 10.3390/neuroglia5040032

**Published:** 2024-12-19

**Authors:** Ki Jung Kim, Rachel E. Patterson, Juan Ramiro Diaz, Philip O’Herron, Weston Bush, Ferdinand Althammer, Javier E. Stern, Michael W. Brands, Zsolt Bagi, Jessica A. Filosa

**Affiliations:** 1 Department of Physiology, Medical College of Georgia, Augusta University, Augusta, GA 30912, USA; 2 Neuroscience Institute, Georgia State University, Atlanta, GA 30302, USA

**Keywords:** hypoperfusion, neurovascular unit, adenosine, astrocyte, calcium

## Abstract

**Background:**

Chronic hypoperfusion is a risk factor for neurodegenerative diseases. However, the sequence of events driving ischemia-induced functional changes in a cell-specific manner is unclear.

**Methods:**

To address this gap in knowledge, we used the bilateral common carotid artery stenosis (BCAS) mouse model, and evaluated progressive functional changes to neurons, arterioles, astrocytes, and microglial cells at 14 and 28 days post-BCAS surgery. To assess the neuro-glio-vascular response to an acute ischemic insult, brain slices were superfused with low O_2_ conditions. Using whole-cell patch-clamp electrophysiology, we measured basic membrane properties (e.g., resting membrane potential, capacitance, input resistance) in cortical pyramidal neurons. The activity of astrocytes was evaluated by monitoring Ca^2+^ from *Aldh1l1-CreERT2*; *R26-lsl-GCaMP6f* mice. Vascular reactivity to low O_2_ from the BCAS mice was also assessed ex vivo.

**Results:**

Our data showed no changes to the basic membrane properties of cortical pyramidal neurons. On the other hand, astrocyte activity was characterized by a progressive increase in the resting Ca^2+^. Notably, at 14 and 28 days post-BCAS, there was an increased expression of anti-inflammatory-related markers (IL-10, S100A10, TRPA1, and Nrf2). These data suggest that, in young mice, BCAS-induced increases in resting Ca^2+^ were associated with the expression of neuroprotective signals. Contrary to observations in glial cells, vascular function was impaired post-BCAS surgery, as shown by a blunted vasodilatory response to low O_2_ and the vasodilatory signal, adenosine.

**Conclusions:**

Together, these data suggest that, in young mice, BCAS leads to vascular dysfunction (e.g., impaired vasodilation in parenchymal arterioles), and in the absence of neuronal dysfunction, mild ischemia is associated with the activation of glial-derived neuroprotective signals.

## 1. Introduction

Cerebral ischemia, whether acute or chronic, can trigger both pro-survival and antisurvival processes [1]. Pro-survival mechanisms, such as those involving nitric oxide, HIF-1alpha, Nrf2, and VEGF, aim at promoting tissue repair and restoring cerebral perfusion [2]. However, deleterious pathways, such as those resulting in calcium (Ca^2+^) dysregulation and cytotoxicity, lead to cell death. The delicate equilibrium of these processes is constrained by the brain’s highly metabolic nature, limited energy reserves, and the duration and intensity of the ischemic insult. Given the enormous detrimental impact of ischemia on cognitive function in pathologies such as stroke, there is a critical need to elucidate the intricate interplay between these pro-survival pathways and those leading to cell death.

Additionally, the different cell types within the neurovascular unit (NVU) exhibit variable resilience to ischemia [3–5]. While neurons are particularly vulnerable, the extent to which other cells like vascular and glia can ameliorate ischemic-induced damage remains poorly understood. Thus, animal models of chronic brain hypoperfusion present a unique opportunity to increase our knowledge of the progression of events associated with ischemia across the cells of the NVU. Understanding these processes will aid in identifying tissue- or cell-specific cellular and molecular targets that can prevent and treat neurodegenerative diseases.

To address the complexity of ischemia-induced changes to critical elements of the NVU, we employed a well-characterized murine model of vascular cognitive impairment (VCI) bilateral common carotid artery stenosis (BCAS). This model induces an abrupt reduction in cerebral blood flow (CBF)~30–40% from baseline, followed by partial restoration [6]. The sequelae established during the ischemic period is poorly understood. As BCAS recapitulates the pathological hallmarks of VCI, such as white matter rarefaction, astrogliosis, microgliosis, inflammation, and cognitive dysfunction [7–9], we evaluated progressive changes to elements of the NVU.

Studies evaluating the impact of BCAS on astrocytes are scarce and mostly limited to their inflammatory state, and within restricted brain regions such as white matter and the hippocampus [8].

Acutely, astrocytes respond to hypoxia with an increase in intracellular Ca^2+^ and, under certain conditions, the release of ATP [10,11]. At the metabolic level, astrocytes can sustain neuronal metabolic activity via the release of lactate from anaerobic glycolysis [12,13]. However, astrocyte homeostatic capacity may be short-lived, causing these cells to transition from a protective (A2) to a pro-inflammatory (A1) phenotype [14,15]. Despite considerable knowledge about astrocyte contributions in severe ischemic events like stroke, their role under mild chronic ischemia remains understudied. Notably, vascular cognitive impairment and dementia (VCID) and Alzheimer’s disease, where chronic hypoperfusion prevails, are linked to alterations in astrocyte Ca^2+^ activity [16–18]. However, the association between astrocyte Ca^2+^ and their phenotype state is poorly understood.

At the vascular level, most cerebral vessels dilate to ischemia [19–22]. However, chronic ischemia can result in oxidative stress and vascular dysfunction [23]. Supporting functional changes to the cerebral microcirculation in BCAS mice, we previously showed that parenchymal arterioles exhibited impaired myogenic (pressure-induced) constrictions 28 days post-BCAS surgery [24]. Others have reported impaired endothelium-dependent dilation [25] and significant reductions in capillary perfusion [26].

In the current study, utilizing an ex vivo preparation that assesses the integral function of the NVU, we explored the concurrent impact of mild chronic hypoperfusion on vascular, astrocytic, and neuronal responses post-BCAS surgery. Our study reveals that, in young mice, chronic hypoperfusion induced changes to vascular and glial cells but not cortical pyramidal neurons.

## 2. Materials and Methods

All experiments were conducted in 8–14 week old male mice, including C57BL6 (Jackson Laboratories, Bar Harbor, ME, USA) and *Aldh1l1-CreERT2*; *R26-lsl-GCaMP6f* mice resulting from the cross-breeding of JAX Stock No. 029655: B6N.FVB-Tg(*Aldh1l1*-cre/ERT2)1Khakh/J and JAX Stock No. 029626: B6N.Cg-Gt(ROSA)26Sor < tm1(CAGGCaMP6f)Khakh > /J R26-Lck-GCaMP6fflox following protocols approved by the animal care and use committees (IACUC) of Augusta University (Protocol #2011-0319). Littermate controls were used for the *Aldh1l1-CreERT2*; *R26-lsl-GCaMP6f* mice experiments. Before experimentation, animals were housed in a room maintained at 20–22 °C with a 12:12 h light–dark cycle and given ad libitum access to food and water.

### 2.1. BCAS and Laser-Doppler Perfusion Imaging

Mice were anesthetized using 2% isoflurane, and an incision was performed to expose the skull. Baseline whole-brain CBF was measured using a Laser Doppler Perfusion Imager (LDPI; Perimed Periscan Pim 3) while maintaining a constant distance (10–10.9 cm) between the LDPI scanner and the mouse skull. Cortical blood flow was measured before introducing micro coils into the common carotid artery and again immediately after BCAS surgery. Following baseline CBF recordings, a small incision was made to expose the common carotid artery. The artery was then carefully separated from the fascia and vagus nerve. After that, the common carotid artery was gently lifted, and a steel micro coil (0.08 mm string diameter, 0.18 mm inner diameter, 0.5 mm pitch, 2.5 mm total length; Wuxi Samini Spring and Sawane Spring) was twined around the artery immediately below the bifurcation of the internal and external carotid arteries. The procedure was then repeated on the contralateral side. Upon termination of the BCAS surgery, the second CBF measurement was performed. The scalp was closed and animals were allowed to fully recover until fully conscious, and then given ad libitum access to food and water. Sham surgeries included all steps, excluding the addition of micro coils. Exclusion criteria: If the surgery was successful, animals were included in the study based on a reduction in CBF with Laser Doppler.

### 2.2. Brain Slice Preparation

Following anesthesia with sodium pentobarbital, the brain was removed, cut into 250–300 μm thick coronal slices using a vibratome (Leica VT 1200S; Leica Microsystems, Wetzlar, Germany), and placed in cold artificial cerebrospinal fluid (aCSF; 3 mM KCl, 120 mM NaCl, 1 mM MgCl_2_, 26 mM NaHCO_3_, 1.25 mM NaH_2_PO_4_, 10 mM glucose, 2 mM CaCl_2_, and 400 μM L-ascorbic acid; osmolarity, 300–305 mOsm), equilibrated with 95% O_2_/5% CO_2_. Slices were kept at room temperature (RT) in aCSF until transferred to the microscope chamber. All experiments were conducted at a chamber temperature of 33° ± 1 °C using a single-line solution heater (SH-27G; Warner Instruments, Hamden, CT, USA) connected to a DC power supply (1735A; BK Precision, Yorba Linda, CA, USA) and continuously perfused with aCSF at a rate of 2–3 mL/min using a peristaltic pump (Miniplus 3; Gilson, Middleton, WI, USA).

### 2.3. Calcium Imaging

Experiments were conducted using the Andor Revolution system (Andor Technology Belfast, UK). A Nikon microscope (Eclipse FN 1, Nikon, Tokyo, Japan) was connected to a laser confocal spinning unit (CSU-X1, Yokogawa, Tokyo, Japan) attached to a Sutter filter wheel and an ultrasensitive EMCCD camera (iXon^EM^, Andor Technology, Belfast, UK) [27]. Calcium imaging was conducted in sham or BCAS (14d or 28d) *Ald1h1-CreERT2*; *R26-lsl-GCaMP6f* mice and visualized using a 40X Nikon objective (NIR Apo, 40X/0.8 w, DIC N2, ∞/0 WD 3.5). Expression of the Ca^2+^ indicator GCaMP6f was induced following five consecutive days of tamoxifen injections (~75 mg/kg). BCAS surgery was conducted following tamoxifen administration. Fluorescence images were obtained using a krypton/argon laser (488 nm excitation and >495 nm emission). Images were acquired at 2 frames/s for ~10 min.

### 2.4. Vessel Cannulation

Arteriole cannulas (ID, 1.17 mm; OD, 1.50 mm; G150TF-3; Warner Instruments) were pulled with a P-97 micropipette puller (Sutter Instruments; Novato, CA, USA), then beveled (KT brown type micropipette beveller BV-10; Sutter instrument) and mounted onto a micromanipulator. Before cannulation, the cannula resistance was determined from a flow–pressure curve, as previously reported [27]. Parenchymal arterioles were visualized using a 60X Nikon (NIR Apo, 60X/1.0 w, DIC N2, ∞/0 WD 2.8) objective with Infrared Differential Interference Contrast (IR-DIC) optics. The luminal flow was controlled with a syringe pump (PHD 2000; Harvard Apparatus, Holliston, MA, USA). A pressure transducer was placed immediately before the cannula for constant pressure monitoring (Servo Pump PS/200; Living System Instrumentation, Burlington, VT, USA). The internal cannula solution consisted of 3 mM KCl, 135 mM NaCl, 1 mM MgCl_2_, 10 mM glucose, 10 mM HEPES, 2 mM CaCl_2_, and 1% albumin [28], with an osmolarity of 300–305 mOsm at pH 7.4 (adjusted with NaOH). The tip of the cannula was maneuvered towards the entrance of the parenchymal arteriole and slowly introduced into the vessel lumen; the step-by-step details of the procedure can be found in [29]. Vascular resistance was determined by subtracting the cannula resistance from the total resistance. The calculated vascular resistance, determined from Ohm’s law, Q = ΔP/R, where Q is flow rate, ΔP is the difference in pressure, and R is resistance (in arbitrary units [AU]), was used to determine the flow rate (Q) required to reach the desired intravascular pressure [29].

### 2.5. Vascular Reactivity Measurements

Studies of flow/pressure-induced myogenic tone in C57BL6 mice were performed in sham, BCAS 14d, and BCAS 28d post-surgery mice. Vascular diameter and lumen pressure values were recorded throughout the experiment at a frequency of 1 image/s using PCO camware and Clampex 9.2 software, respectively. Upon cannulation, the arteriole flow rate was set to induce a starting lumen pressure of ~30–40 mmHg (estimated physiological pressure for a parenchymal arteriole); these conditions were sustained until a plateau was established. After reaching a plateau, arterioles were exposed to a stimulus or agonist. To obtain comparable levels of baseline tone across groups, experiments measuring the parenchymal arterioles responses to adenosine were conducted at a low-pressure value of ~15 mmHg and the arterioles were further constricted via bath application of U46619 (50–100 nM). Using this protocol, we could overcome the between-group variability in resting tone resulting from chronic hypoperfusion [24]. At the end of the experiment, slices were perfused with zero Ca^2+^ aCSF containing 100 μM papaverine to obtain the maximum diameter (100%) at each flow rate used for each protocol. Diameter values are expressed as %tone relative to the average maximum diameter at each flow rate. For percent relaxation, tone was calculated relative to baseline tone.

### 2.6. Electrophysiology

Whole-cell currents were obtained using a Multiclamp 700B amplifier (Axon Instruments, Foster City, CA, USA). Patch pipettes were made from thin-walled borosilicate glass (OD, 1.5 mm; ID, 0.86 mm; Sutter instrument BF150-86-7.5) and pulled using a P-97 puller (Sutter Instruments) to a resistance of 4–6 MΩ. The internal solution consisted of 130 mM K-gluconate, 10 mM HEPES, 10 mM BAPTA, 10 mM KCl, 0.9 mM MgCl_2_, 4 mM Mg_2_ATP, 0.3 mM Na_2_GTP, and 20 mM phosphocreatine, with an osmolarity of 291–295 mOsm at pH 7.2 (adjusted with KOH). A square-pulse protocol was used to determine whether the recording neuron showed spike frequency adaptation, indicative of a pyramidal neuron, as previously described [30]. For current-clamp mode recordings and to generate neuronal action potentials (AP), neurons were brought to near spike threshold (approximately −45 mV) by injecting a depolarizing current and holding it until a stable AP frequency was established. The current signals were filtered with a 1 kHz low-pass filter and digitized at 10 kHz using a Digidata 1322A acquisition system (Axon Instruments). The input–output function was assessed by subjecting patched neurons to depolarizing steps of increasing magnitude (0–300 pA) and plotting the number of evoked spikes as a function of depolarizing steps. The voltage output was digitized at a 16-bit resolution at 10 kHz and filtered at 2 kHz. pClamp10.6 (Axon Instruments) was used for data acquisition and storage. Only neurons with a stable baseline firing activity following depolarizing DC current injections were used.

### 2.7. Immunohistochemistry

Brains from sham, BCAS 14d, and BCAS 28d mice (C57BL6) were fixed in 4% paraformaldehyde for 48 h at 4 °C and then transferred to 0.01 M PBS containing 30% sucrose for 72 h. Brains were stored at −80 °C until sectioning. Using a Leica CM3050 S cryostat (Leica Microsystems, Wetzlar, Germany), 50 μm coronal sections were obtained and stored in a cryoprotectant solution (50 mmol/L PBS, 30% ethylene glycol, 20% glycerol). Before staining, the cryoprotectant solution was removed by washing the tissue with 0.01 M PBS. Fixed slices were then blocked for one hour in 0.01 M PBS containing 0.3% Triton X-100, 0.04% NaN_3_, and 10% horse serum (Vector Labs, Burlingame, CA, USA). Slices were then incubated at room temperature for 48 h in Iba-1 anti-rabbit (1:1000, Wako, Richmond, VA, USA), suspended in 0.3% Triton X-100 0.01 M PBS. Following corresponding washes in 0.01 M PBS, slices were incubated for four hours in Alexa 594 donkey anti-rabbit (1:250, Jackson Immuno Research, West Grove, PA, USA) also in 0.3% Triton X-100 0.01 M PBS. Slices were then washed three times with 0.01 M PBS and subsequently mounted using Vectashield (Vector Labs Burlingame, Newark, CA, USA). Image acquisition was conducted using a confocal microscope (Zeiss LSM 510, Jena, Germany) equipped with a 40× oil immersion objective and Zen 2012 SP1 acquisition software. We acquired 1 μm interval Z-stacks from cortical layers III-V in both hemispheres.

### 2.8. Reverse Transcription Polymerase Chain Reaction (RT-PCR) and Quantitative Real-Time PCR (qPCR) from Brain Region-Specific Tissue Punches

RNA extraction and isolation were carried out using the miRNAeasy Mini Kit (Qiagen (Hilden, Germany), Cat. No. 217004) and the QIAzol Lysis Reagent (Qiagen, Mat. No. 1023537) following the manufacturer’s instructions. Tissue sections, 100 μm thick, were prepared using a cryostat (Leica CM3050S, Wetzlar, Germany) at −20 °C. Punches were taken from the somatosensory cortex S1BF (Bregma −0.4 mm to Bregma −1.5 mm) bilaterally, using a 0.75 mm tissue puncher. The samples were stored on dry ice until RNA extraction. RNA concentrations, ranging from 145 to 220 ng/μL, were measured with the NanoDrop One (Thermo Scientific, Waltham, MA, USA) before cDNA synthesis. cDNA was synthesized using the iScriptTM gDNA Clear cDNA Synthesis Kit (BIO RAD, Cat. No. 1725035, Hercules, CA, USA) and the SimpliAmp Thermal Cycler (Thermo Fisher Scientific) following the manufacturer’s protocol. qPCR was performed using the Roche LightCycler96 with the standard 45-cycle amplification protocol. QuantiTect primers (Qiagen), diluted to a final concentration of 200 nM in 1.1 mL TE buffer (pH 8.0), were used. Each qPCR reaction (brain region, primer, and condition) was run in triplicate, and the average values were calculated for statistical analysis.

### 2.9. Data Analysis

Electrophysiology data were analyzed using Clampfit 10.6 (Axon Instruments). The firing discharge was recorded in current-clamp mode. The mean frequency was obtained before, during, and after bath application of the stimulus (20% O_2_). Cell capacitance was calculated by integrating the area under the transient capacitive phase of a 5 mV depolarizing step pulse (voltage-clamp mode). To detect Ca^2+^ events in brain slices from *Ald1h1-CreERT2*; *R26-lsl-GCaMP6f* mice, we used *AQuA* and Matlab R2023b [31].

GraphPad Prism 10 software (GraphPad Software, La Jolla, CA, USA) was used for all statistical analyses. Values are expressed as means ± S.E.M. Differences between groups were determined using a one- or two-way repeated measures analysis of variance (ANOVA) with corresponding multiple-comparison post hoc tests, as specified in figure legends. Statistical significance was accepted at the 95% (*p* < 0.05) confidence level, and individual *p*-values, determined from multiple comparison tests, are indicated in the text and figure legends.

## 3. Results

### 3.1. Ischemia-Induced Pyramidal Neuron Responses in BCAS Mice

**Figure 1.**
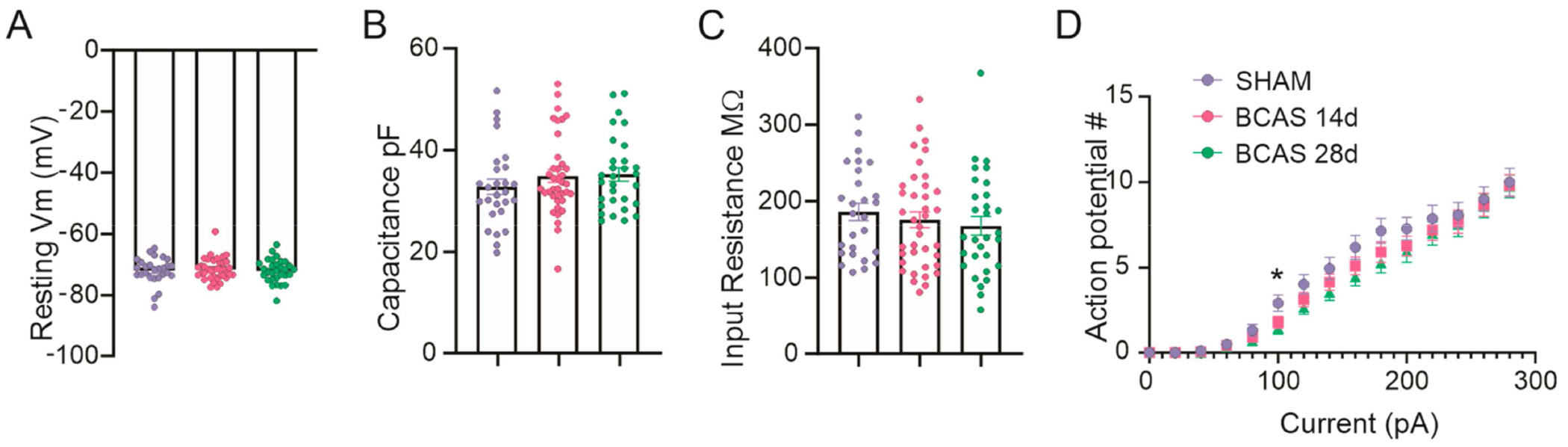
Basic membrane properties from cortical pyramidal neurons post-BCAS surgery. Whole-cell patch-clamp recordings from sham, BCAS 14d, and BCAS 28d neurons. (**A**) Summary data corresponding to resting membrane potential. (**B**) Summary data corresponding to cell capacitance. (**C**) Summary data corresponding to input resistance. (**D**) Input–output function. One-way ANOVA followed by Dunnett’s multiple comparison test (MCT) (*n* = 27 neurons for sham, *n* = 38 neurons for BCAS 14d, and *n* = 30 neurons for BCAS 28d). Data expressed as mean SEM, * *p* < 0.05 vs. sham.

To address the impact of chronic hypoperfusion on neuronal function post-BCAS surgery, we used whole-cell patched-clamp recordings to assess basic membrane properties and the input–output function of cortical pyramidal neurons from sham, 14, and 28 days post-BCAS surgery mice (or BCAS 14d and BCAS 28d, respectively). Chronic hypoperfusion did not affect the basic membrane properties (Figure 1A–C) or the input–output function of cortical pyramidal neurons (Figure 1D).

**Figure 2.**
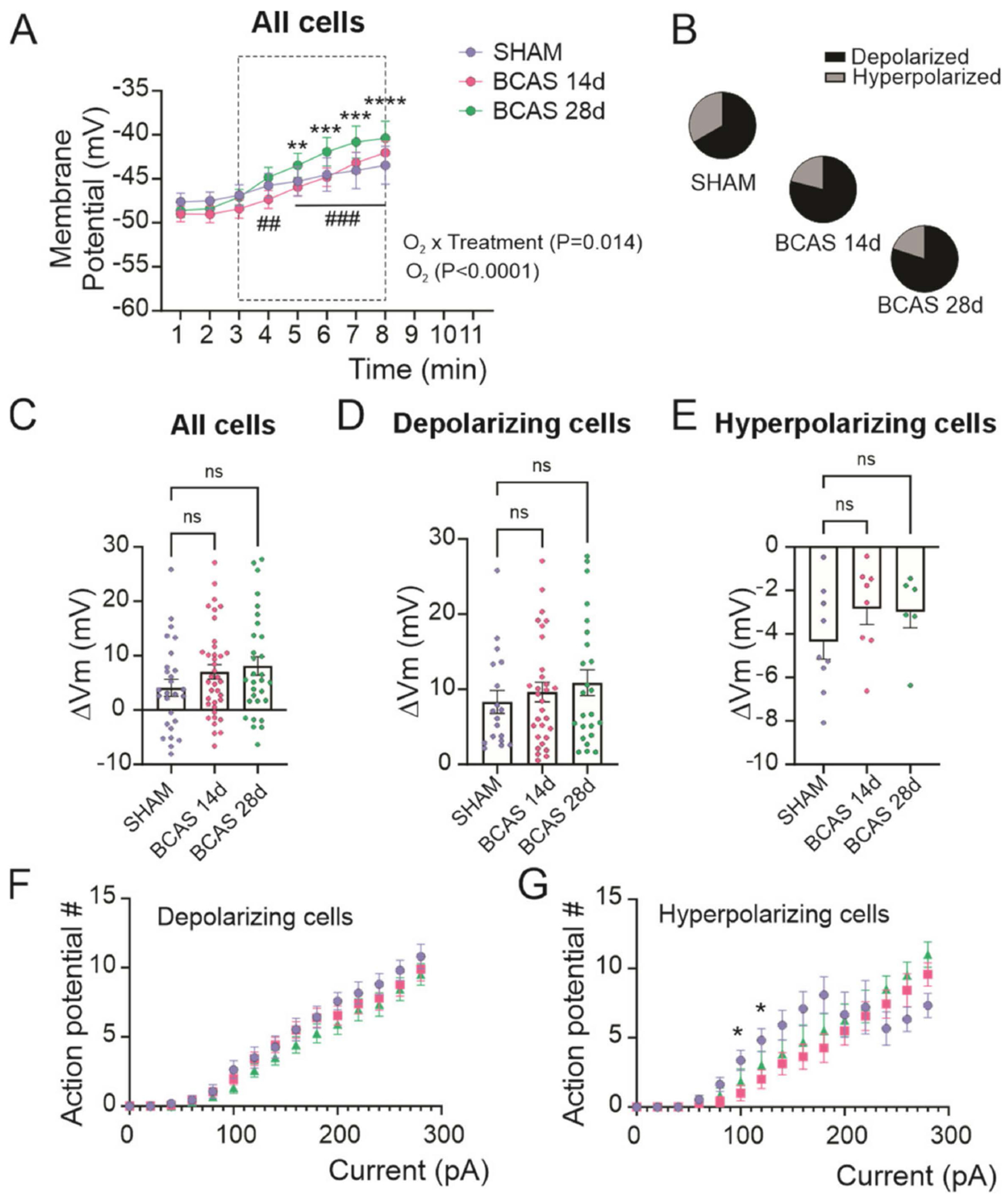
Low O_2_-induced changes in membrane properties of cortical pyramidal neurons from sham, 14, and 28 days post-BCAS surgery mice. (**A**) Low O_2_-induced changes in resting membrane potential. (**B**) Proportion of neurons showing depolarization vs. hyperpolarization in response to low O_2_. (**C**–**E**) Delta membrane potential resulting from low O_2_ exposure to sham, BCAS 14d, and BCAS 28d brain slices. (**F**,**G**) Number of action potentials (AP) at various step currents from depolarizing (K) and hyperpolarizing (L) cortical neurons. (**A**,**B**) Two-way ANOVA followed by Sidak’s MCT (n-27 sham, *n* = 38 BCAS 14d, *n* = 30 BCAS 28d). (**C**–**E**) One-way ANOVA, followed by Dunnett’s MCT (C, *n* = 27 sham, *n* = 38 BCAS 14, *n* = 30 BCAS 28d; D/E, *n* = 18/9 sham, *n* = 30/8 BCAS 14, *n* = 24/6 BCAS 28d) (**F**,**G**) The mixed-effects model was followed by Dunnett’s MCT (*n* = 16/11 sham, *n* = 30/8 BCAS 14d, *n* = 30/6 BCAS 28d). Data expressed as mean SEM. * *p* < 0.05, ** or ^##^ *p* < 0.01, *** or ^###^ *p* < 0.001, **** *p* < 0.0001, ns = not significant. Symbols for groups showing significances are * BCAS 14d, and ^#^ BCAS 28d.

The brain of chronically hypoperfused mice may experience episodic ischemic events, increasing neuronal vulnerability. Thus, we measured the acute response of the cortical pyramidal neurons to low O_2_ by switching the perfusate (aCSF) from 95% O_2_/5% CO_2_ to one gassed with 20% O_2_/5% CO_2_/balanced N_2_. On average, an acute (5 min) low O_2_ stimulus evoked a membrane depolarization in all groups, including sham, BCAS 14d, and BCAS 28d (low O_2_ x treatment, *p* < 0.014), as shown in Figure 2A. Within-group comparisons revealed significant changes from the baseline for BCAS 14d (*p* < 0.005) and BCAS 28d (*p* < 0.005) neurons, but not for sham. All groups revealed mixed neuronal responses characterized by an ischemia-induced depolarization or an ischemia-induced hyperpolarization. The proportion of neurons responding with depolarizations was greater in BCAS mice (Figure 2B). We compared the low O_2_-evoked membrane potential changes in all neurons (Figure 2C), in depolarizing neurons (Figure 2D) and in hyperpolarizing neurons (Figure 2E). There were no differences between groups. In addition, there were no differences in the input–output response for depolarizing (Figure 2F) or hyperpolarizing neurons (Figure 2G).

Collectively, these data suggest that, at baseline, the basic neuronal properties were comparable between groups. In addition, while the number of depolarizing neurons to the acute low O_2_ stimulus was greater in BCAS mice, the averaged response was not affected by the BCAS surgery.

### 3.2. Reduced Hypoxia-Evoked Vascular Responses in BCAS Mice

**Figure 3.**
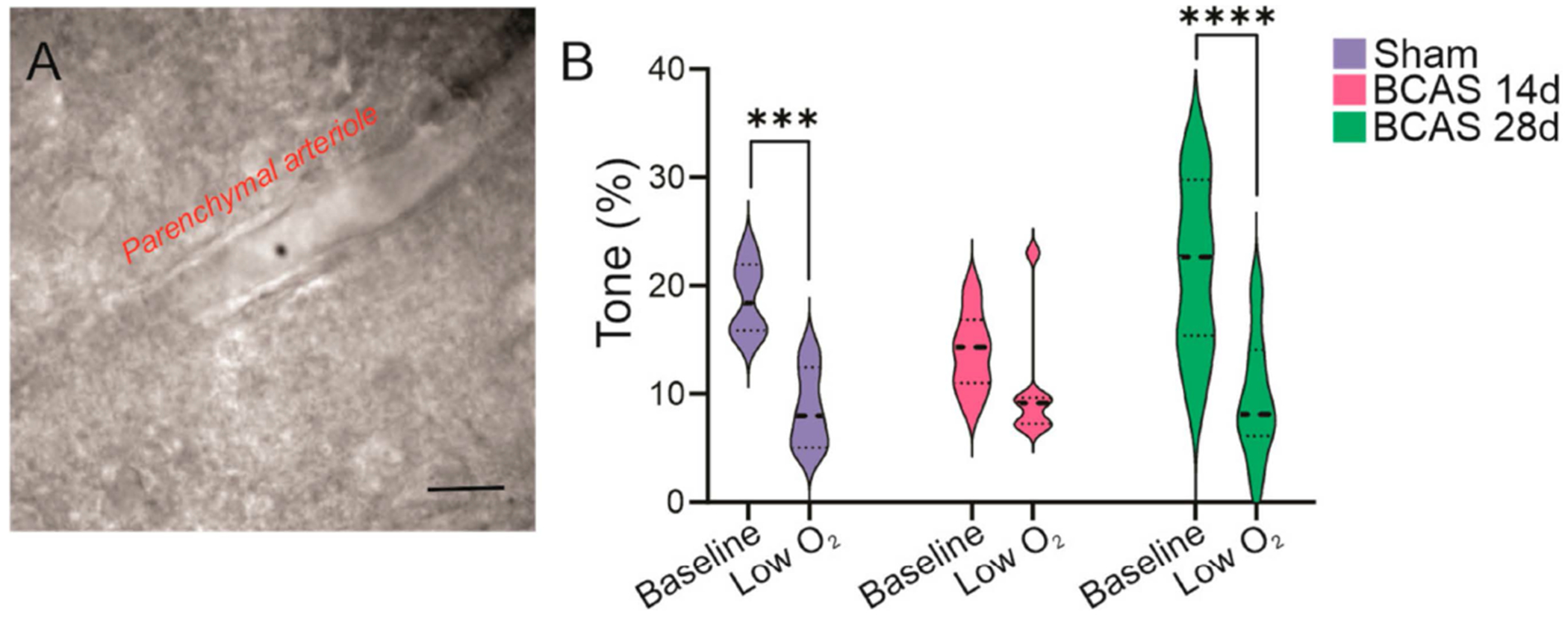
Low O_2_-induced changes in parenchymal arteriole vasoreactivity. (**A**) Differential interference contrast image of a cannulated and perfused parenchymal arteriole in a brain slice preparation. (**B**) Vascular reactivity to bath applied low O_2_ treatment in sham, BCAS 14d, and BCAS 28d mice. Two-way ANOVA followed by Sidak’s MCT (*n* = 6 sham, *n* = 7 BCAS 14d, *n* = 7 BCAS 28d). Data expressed as mean SEM. *** *p* < 0.001 and **** *p* < 0.0001. Scale bar = 20 μm.

Using an ex vivo brain slice preparation that incorporates flow and pressure within an arteriole in a brain slice [27], we measured parenchymal arteriole reactivity to low O_2_ in sham, BCAS 14d, and 28d post-surgery mice. A cortical arteriole was cannulated and pressurized to ~30–40 mmHg, as previously described [24]. Following an equilibration period and tone development, the brain slice was exposed to 20% O_2_ (5 min), and vessel diameters were measured over time. A significant decrease in vascular tone was observed in arterioles from sham (*p* = 0.0006) and BCAS 28d (*p* < 0.0001) mice. On the other hand, arterioles from BCAS 14d mice did not show reactivity to low O_2_ (Figure 3).

### 3.3. Reduced Adenosine-Evoked Relaxation in BCAS Mice

**Figure 4.**
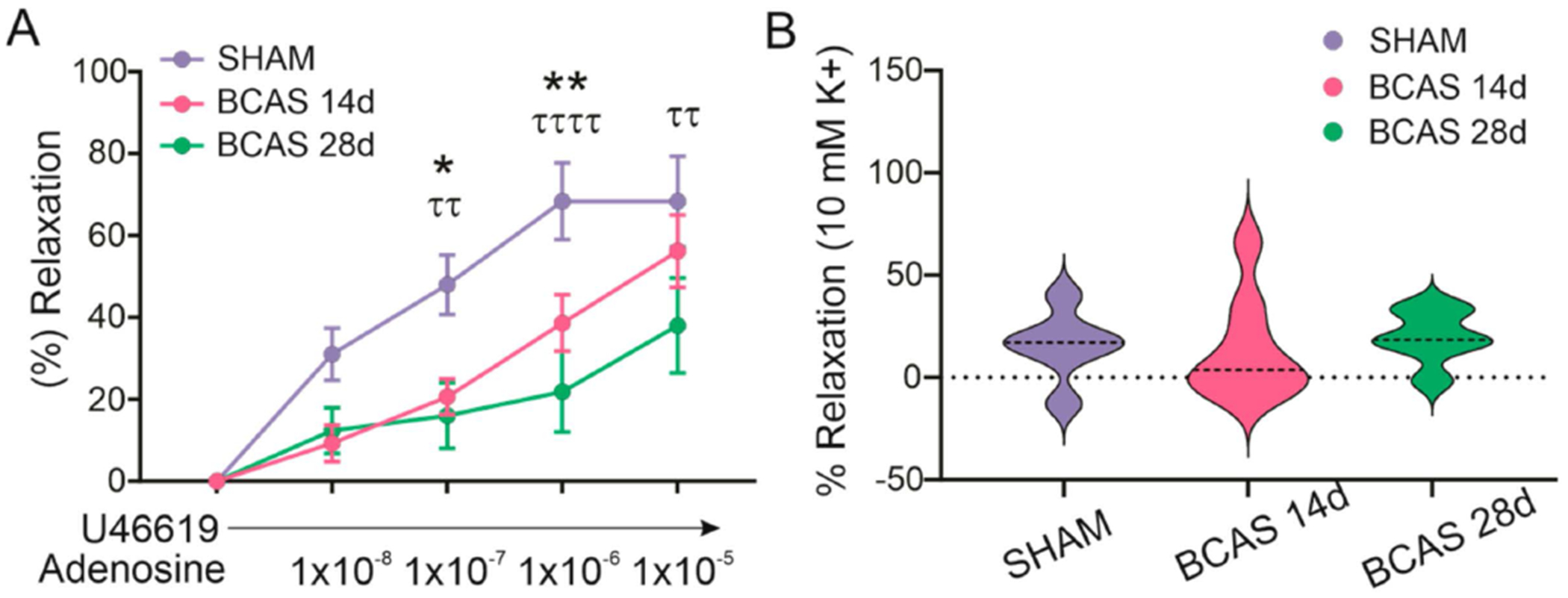
Parenchymal arteriole responses to adenosine and 10 mM K^+^. (**A**) Summary data showing percent (%) relaxation of pressurized parenchymal arterioles responses to increasing concentrations of adenosine in sham (*n* = 6), BCAS 14d (*n* = 8), and BCAS 28d (*n* = 7) mice. Two-way ANOVA followed by Dunnett’s MCT (between group comparisons * sham vs BCAS 14d, τ sham vs. BCAS 28d). (**B**) Summary data showing percent (%) relaxation of pressurized parenchymal arterioles to K^+^ in sham (*n* = 6), BCAS 14d (*n* = 8), and BCAS 28d (*n* = 6) mice. One-way ANOVA followed by Dunnett’s MCT vs. sham. Data expressed as means ± SEM. * *p* < 0.05, **^/ττ^ *p* < 0.01 and ^ττττ^ *p* < 0.0001.

Ischemia leads to the release of adenosine, which not only suppresses neuronal activity [32] but can evoke vasodilation [33]. In arterioles, adenosine 2 receptor (A_2_R) activation is associated with vasodilation, whereas A_1_R activation mediates vasoconstriction [33,34]. We next tested parenchymal arteriole reactivity to increasing concentrations of bath-applied adenosine (10 nM–10 μM). In response to adenosine, parenchymal arterioles from sham mice progressively dilated; significant differences were observed at all concentrations compared to baseline tone. In BCAS 14d, no differences were observed at the lowest adenosine concentration (10 nM), but significant relaxations were observed at all other concentrations. On the other hand, in BCAS 28d, relaxations were only significant at the highest concentration (10 μM) (*p* = 0.05), Figure 4. When we compared the percent relaxation between groups at each concentration, we observed a significant reduction in the BCAS 14d and BCAS 28d mice at concentrations of 1 × 10^−7^ M (*p* = 0.02 and *p* = 0.006 respectively) and 1 × 10^−6^ M (*p* = 0.008 and *p* < 0.0001), and BCAS 28d at a concentration of 1 × 10^−5^ M (*p* = 0.009). Together, these data show that arterioles in BCAS mice have reduced sensitivity to adenosine.

Potassium is a key signal contributing to neurovascular coupling-mediated vasodilation [35]. In all groups, 10 mM K^+^ evoked vasodilation (15.8 ± 7 for sham, 14.14 ± 8.96 for BCAS 14d, and 19.78 ± 5.317 for BCAS 28d); these were not statistically different between groups (Figure 4B). Together, these data support a blunted arteriole response to low O_2_ and the ischemia-derived signal adenosine in chronically hypoperfused mice. However, no differences in response were observed to the neurovascular coupling signal K^+^.

### 3.4. Spontaneous and Stimulus-Induced Astrocyte Ca^2+^ Dynamics

**Figure 5.**
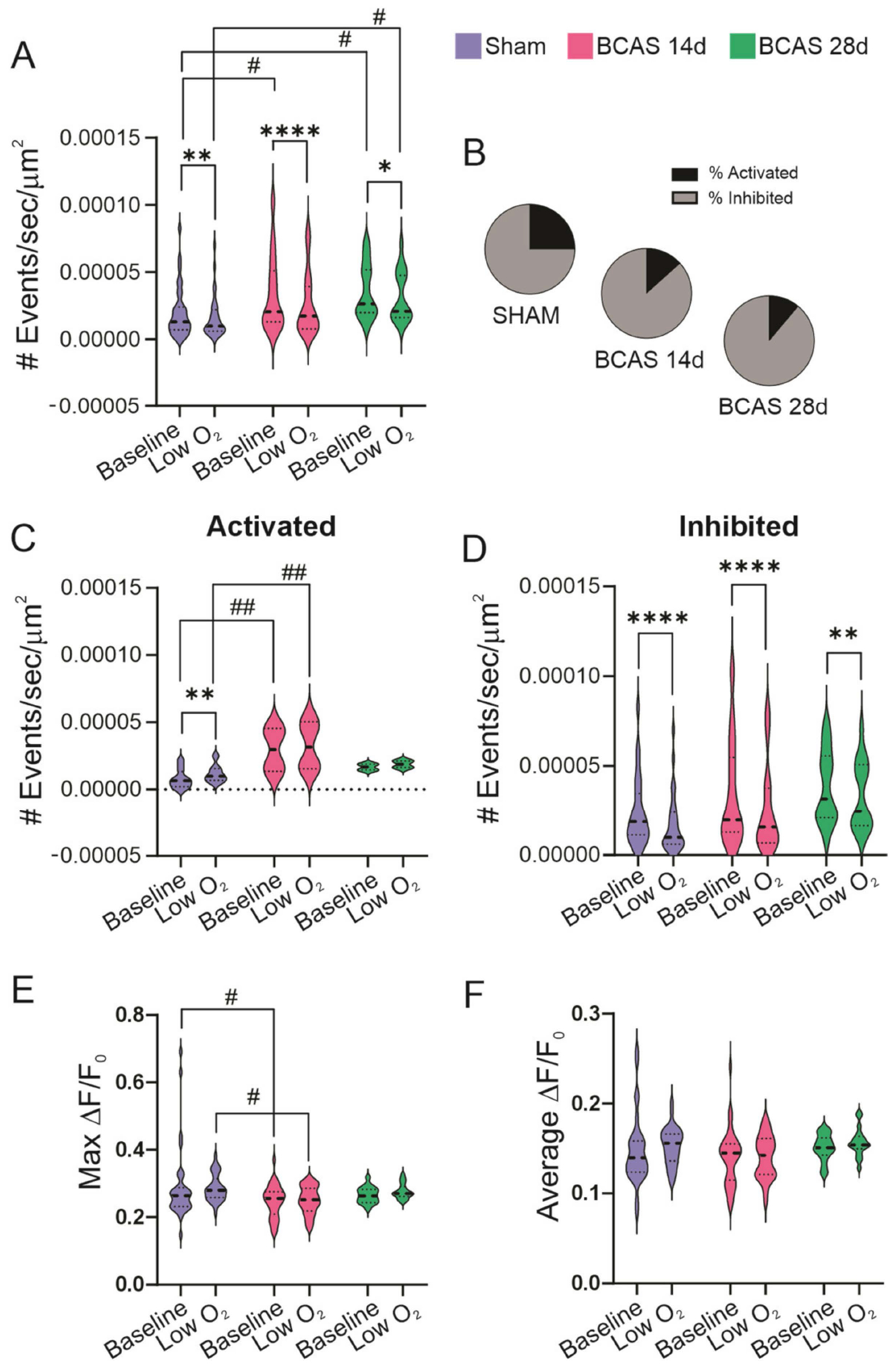
Spontaneous and low O_2_-induced cortical astrocytic Ca^2+^ dynamics post-BCAS ex vivo. (**A**) Summary data showing astrocyte Ca^2+^ events in response to low O_2_ in sham, BCAS 14d, and BCAS 28d mice. (**B**) Proportion of astrocytes responding with an activation or inhibition of Ca^2+^ events to low O_2_. (**C**,**D**) Summary data showing low O2-induced changes in Ca^2+^ events for activated (**C**) and inhibited (**D**) astrocytes. (**E**,**F**) Summary data showing maximum delta F/F_0_ (**E**) and average delta F/F_0_ (**F**). (**A**,**B**,**E**,**F**) Two-way ANOVA followed by Sidak’s MCT (sham (*n* = 32), BCAS 14d (*n* = 30) and BCAS 28d (*n* = 18)). (**C**,**D**) Two-way ANOVA followed by Sidak’s MCT (sham (*n* = 9/23), BCAS 14d (*n* = 10/19) and BCAS 28d (*n* = 6/12)). Data expressed as mean SEM. * or ^#^ *p* < 0.05, ** or ^##^ *p* < 0.01 and **** *p* < 0.0001. (*) Within-group comparisons, (#) between-group comparisons.

In general, acute ischemic events such as stroke, characterized by a complete loss of CBF, increase astrocyte Ca^2+^ [36]. However, the effect of mild ischemia, associated with chronic hypoperfusion, on cortical astrocyte Ca^2+^ signaling is less understood. Using cortical brain slices from sham and BCAS (14d or 28d post-surgery) *Ald1h1-CreERT2*; *R26-lsl-GCaMP6f* mice, we determined whether BCAS altered spontaneous and stimulus-induced Ca^2+^ dynamics in astrocytes. To mimic an acute ischemic event, the bath-applied aCSF was switched from 95%O_2_–5%CO_2_ to 20%O_2_–5%CO_2_. In response to low O_2_, the number of astrocytes Ca^2+^ events/s/area (μm^2^) significantly decreased for sham (*p* = 0.008), BCAS 14d (*p* < 0.0001), and BCAS 28d (*p* < 0.0425) mice. Between-group comparisons revealed a significantly higher baseline activity (vs sham) for BCAS 14d (*p* = 0.0235) and BCAS 28d (*p* = 0.0289). The between-group comparison of the low O_2_ response was significantly higher in BCAS 28d vs. sham (*p* = 0.026). Thus, while acutely, low O_2_ reduced the overall number of astrocytes Ca^2+^ events, the number of events at baseline between groups was progressively higher in the BCAS mice compared to sham (Figure 5A). These data suggests that chronic hypoperfusion progressively increased resting astrocyte Ca^2+^.

Interestingly, the proportion of astrocyte-related events responding with an activation (increased Ca^2+^) vs. inhibition (decreased Ca^2+^) was greater in sham mice than BCAS mice (Figure 5B). We thus compared the magnitude of these responses in low O_2_-induced activated vs. inhibited astrocytes (Figure 5C,D). For activated astrocytes, low O_2_ induced a significant increase in the number of Ca^2+^ events/s/area in sham mice only (*p* = 0.004). Notably, the activity of BCAS 14d was significantly higher both at baseline (*p* = 0.0064) and in the presence of low O_2_ (*p* = 0.0089), when compared to sham mice (Figure 5C). For inhibited astrocytes, all groups showed a significant reduction in the number of Ca^2+^ events/s/area in response to low O_2_ (*p* < 0.0001 for sham, *p* < 0.0001 for BCAS 14d, and *p* = 0.0079 for BCAS 28d), as shown in Figure 5D. The peak amplitude of the Ca^2+^ events (Max ΔF/F_0_) at baseline and in the presence of low O_2_ was greater in sham vs. BCAS 14d (Figure 5E). There were no differences between groups in the average ΔF/F_0_ (Figure 5F).

**Figure 6.**
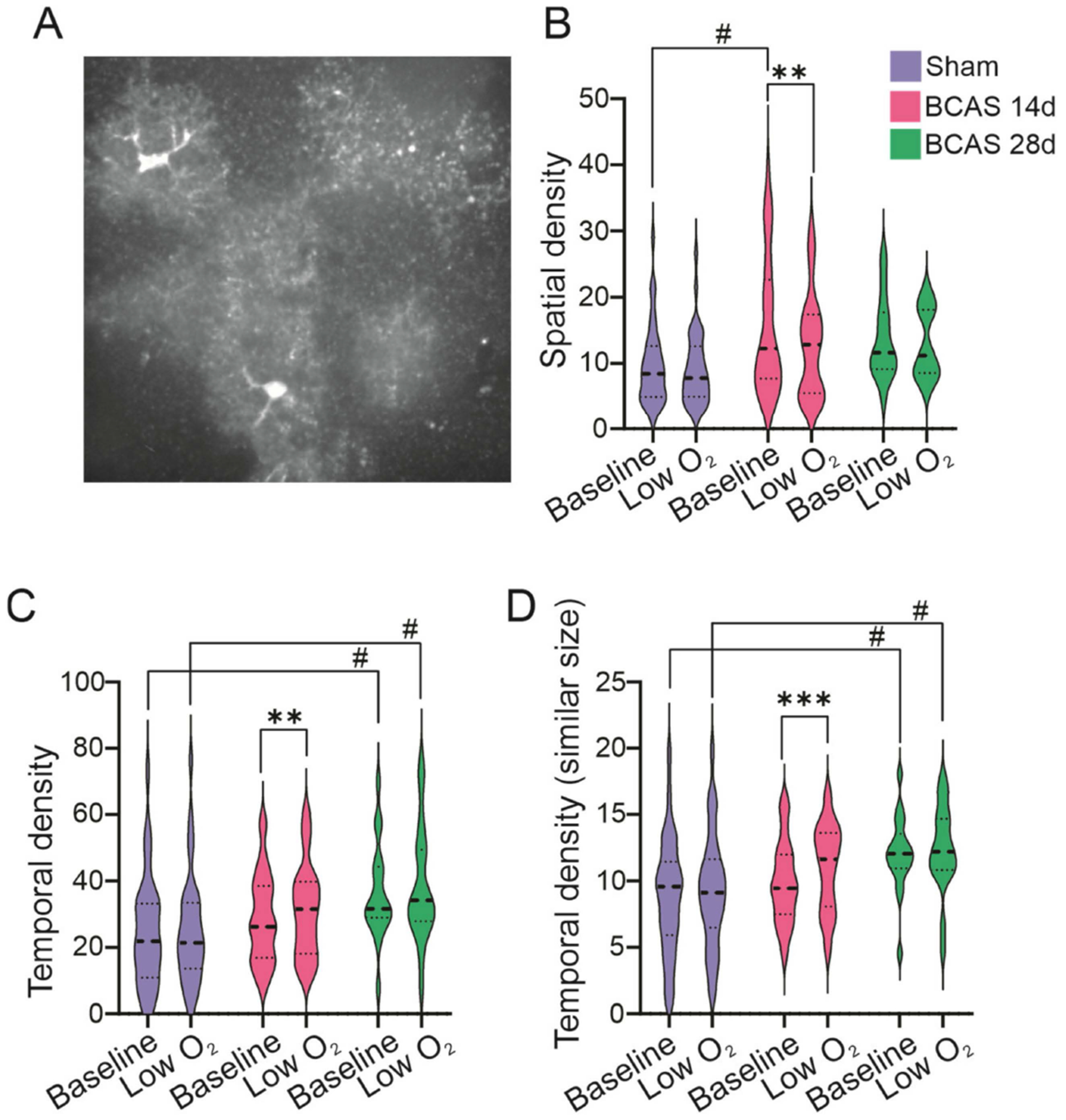
Astrocyte network Ca^2+^ activity changes in response to low O_2_ post-BCAS surgery. (**A**) Representative confocal image of multiple GCaMP6f labeled astrocytes in a brain slice. (**B**) Summary data showing spatial density changes before and after low O_2_ treatment. (**C**,**D**) Summary data showing temporal density (**C**) and temporal density with similar size events (**D**) before and after low O_2_ treatment in sham (*n* = 32), BCAS 14d (*n* = 29), and BCAS 28d (n-18) mice. Two-way ANOVA followed by Sidak’s MCT. Data expressed as means ± SEM. ^#^ *p* < 0.05, ** *p* < 0.01, and *** *p* < 0.001. (*) Within-group comparisons, (#) between-group comparisons.

The *AQuA* software package [31] assesses network dynamics using the spatial and temporal density of the Ca^2+^ events. The spatial density corresponds to the number of events co-occurring with the current event, and the temporal density relates to the number of events that share a spatial footprint with the current event. At baseline, BCAS 14d had a significantly larger spatial density (*p* = 0.0048) vs. sham. The spatial density significantly decreased in response to low O_2_ only in BCAS 14d (*p* = 0.002), Figure 6. At baseline, we also observed a significantly higher temporal density in BCAS 28d vs. sham (*p* = 0.024 and *p* = 0.0064 for similar size events). In response to low O_2_, the temporal density was significantly increased only in the BCAS 14d group (*p* = 0.002 and *p* = 0.0005 for similar size events).

Together, these data support dynamic Ca^2+^ activity changes in the astrocyte network with disease progression. Chronic ischemia leads to a progressive increase in the frequency of astrocyte Ca^2+^ events at baseline and in the network activity dynamics. On average, acute ischemia causes a decrease in the frequency of Ca^2+^ events.

### 3.5. Increased Anti-Inflammatory Markers in Astrocytes and Microglia from BCAS Mice

To assess the impact of chronic hypoperfusion on the neuro-glial inflammatory process, we assessed the structural properties of cortical microglial cells and the expression of cytokine markers from sham, BCAS 14d, and BCAS 28d brains.

**Figure 7.**
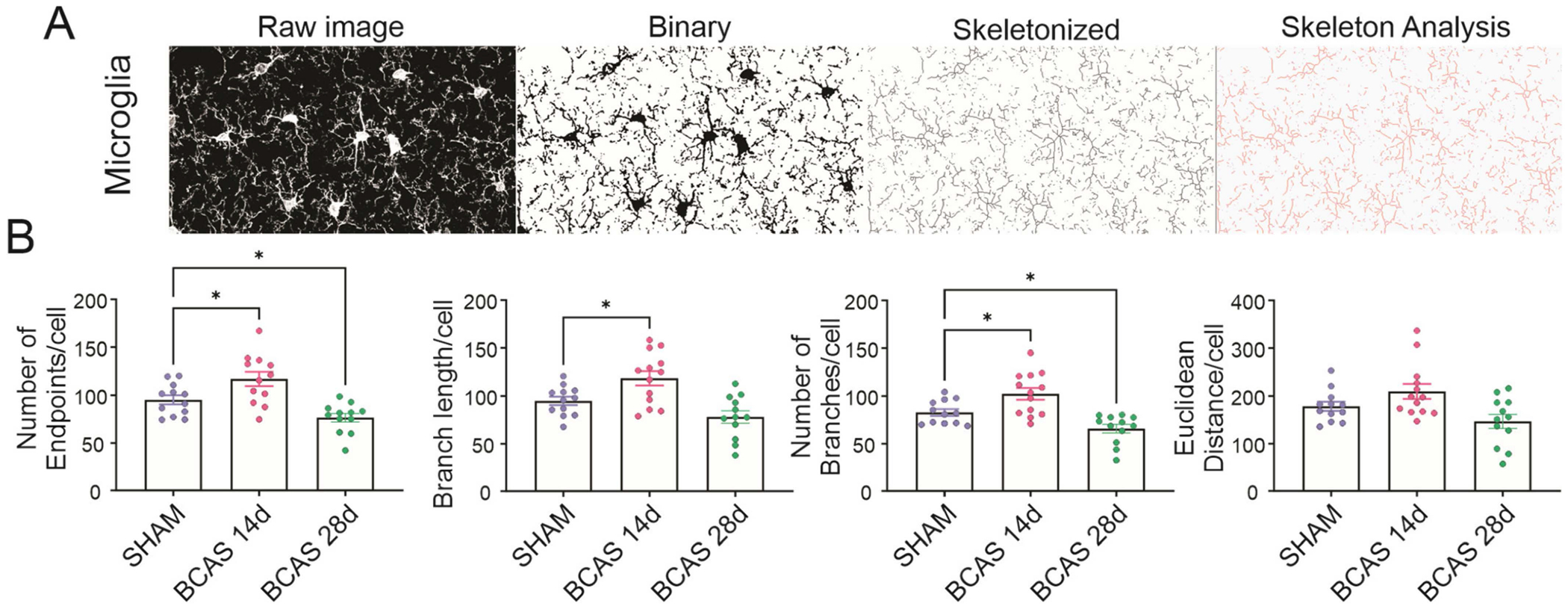
Microglia morphological changes post-BCAS surgery. (**A**) Representative immunofluorescence confocal images of cortical microglia labeled with Iba1 before and after skeleton analysis used for structural quantification. (**B**) Summary data showing the quantification of microglia arborization properties in sham (*n* = 12), BCAS 14d (*n* = 12), and BCAS 28d (*n* = 12) mice brain slices. One-way ANOVA followed by Holm–Sidak’s MCT. Data expressed as means ± SEM. * *p* < 0.05.

Microglial cells are known to change their arborization upon acquiring a reactive phenotype [37]. Increased microglial reactivity changes the rate of processes protrusion/retraction. In general, microglia switching towards a pro-inflammatory state will retract their processes, reflected by a decrease in the number of endpoints obtained from a skeletal analysis [38]. We quantified microglia skeletal properties from sham, BCAS 14d, and BCAS 28d mouse brains. On day 14 post-BCAS surgery, microglia cells appeared more arborized than in sham. On the other hand, on day 28 post-surgery, arborization was significantly decreased, as shown in Figure 7A,B.

**Figure 8.**
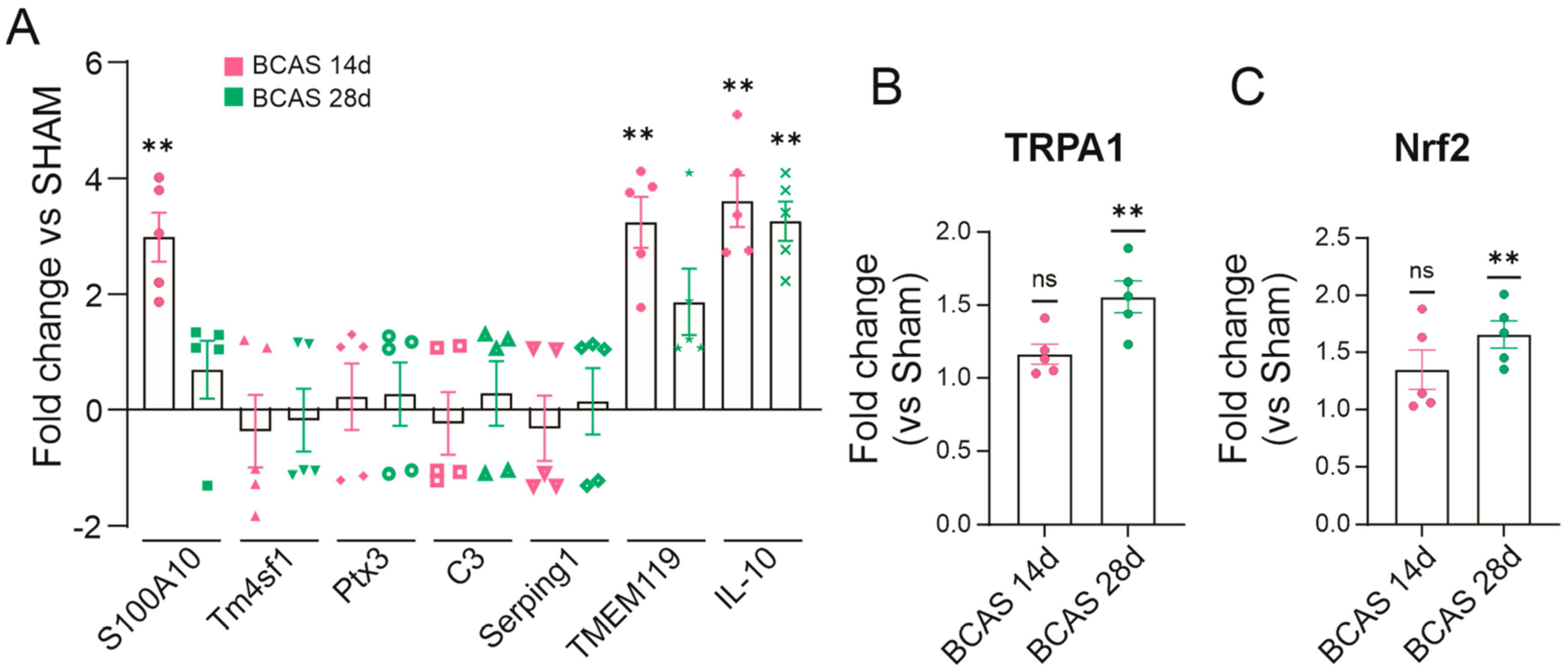
Quantitative mRNA expression of inflammatory markers in BCAS brains. (**A**) Summary data showing fold changes in various inflammatory markers for astrocytes and microglia. (**B**) Summary data showing fold changes in TRPA1 expression level. (**C**) Summary data showing fold changes in Nrf2 expression level. One sample t and Wilcoxon test (*n* = 5 per group). Data expressed as means ± SEM. ** *p* < 0.014. ns = not significant.

While numerous studies have reported changes in the expression of inflammatory markers in glial cells, their association with functional states (e.g., Ca^2+^ dynamics) is limited. To determine if astrocytes and microglia from the BCAS mice were predominantly associated with a pro- or anti-inflammatory state, we quantified key glial markers. Contrary to our hypothesis of increased pro-inflammation, the expression of anti-inflammatory glial markers was significantly increased in both the BCAS 14d and BCAS 28d brains. In the BCAS 14d brains, we observed increased expression of the astrocyte neuroprotective marker S100A10 (*p* = 0.0094), IL-10 (*p* = 0.0044), and microglia TMEM119 (*p* = 0.0044) [39]. The expression of IL-10 (*p* = 0.0027) remained significant in BCAS 28d when compared to sham brains, as shown in Figure 8A. Consistent with the notion that in our model, BCAS showed increased expression of anti-inflammatory markers, we looked for two other putative anti-inflammatory markers known to be affected by ischemia, namely, TRPA1 ion channels [40] and Nrf2 [41]. Ischemia significantly increased TRPA1 and Nrf2 in the BCAS 28d brains but not BCAS 14d (vs sham), as shown in Figure 8B.

## 4. Discussion

Our study demonstrates that BCAS-induced chronic hypoperfusion leads to functional changes in the vascular and glial cells of the neurovascular unit (NVU), but does not significantly affect the basic membrane properties of cortical pyramidal neurons. Specifically, we report impaired stimulus-induced vasodilatory function in cortical parenchymal arterioles, increased astrocyte Ca^2+^ activity, and changes in microglia structure. In young mice, ischemia-induced increases in glial cell reactivity may be linked to the activation of neuroprotective mechanisms.

### 4.1. Ischemia-Induced Changes to Parenchymal Arterioles

Chronic hypoperfusion leads to adaptive changes in the cerebral vasculature that aim to restore CBF. In BCAS, these have been attributed to a reduction in arteriole tone [24] and compensatory mechanisms such as angiogenesis or vessel dilation [42]. If significant, however, ischemia can lead to oxidative stress and vascular dysfunction [8,25]. Our study supports functional changes to parenchymal arterioles. We observed a significant decrease in the arteriole vasodilatory response to low O_2_ and the ischemia-induced vasodilatory signal adenosine. Impaired vascular function may compromise on-demand CBF responses corresponding to changes in metabolic activity. Thus, these functional vascular changes increase brain vulnerability to ischemia.

### 4.2. Dynamic Changes in Astrocytes

Astrocytes are essential players in multiple homeostatic brain functions, including metabolism, the regulation of CBF, and neuronal activity [43]. Astrocytes regulate CBF through Ca^2+^ signaling and the release of vasoactive substances such as K^+^, adenosine, prostaglandins, and eicosanoids [44]. Notably, CBF regulation is critical for neuronal survival, a particularly significant process under hypoxic conditions. Under severe ischemic conditions such as stroke, astrocytes undergo extensive functional and structural changes, which can result in the induction of neuroprotective or pro-inflammatory pathways [45]. Less is known, however, about the role of astrocytes during mild chronic ischemic conditions. However, because astrocytes can sense PO_2_ levels lower than 17 mmHg, a response characterized by increased Ca^2+^ [46], we predicted that BCAS-induced reductions in CBF would increase spontaneous astrocyte Ca^2+^. As shown in Figure 2, Ca^2+^ dynamics in the astrocytes of BCAS mice were significantly higher than in the control group, indicating enhanced reactivity of astrocytes under chronic hypoperfusion. This, however, was not reflected in the F/F_0_, suggesting an increase in the number but not in the magnitude of the Ca^2+^ events. High resting Ca^2+^ may lower the dynamic range and blunt an overall response. In our study, only a proportion of astrocytes from sham mice responded to acute low O_2_ with increased frequency. In BCAS 14d, Ca^2+^ events were already significantly higher. While low O_2_ further increased the number of Ca^2+^ events above those in the sham group, the response was not significant within the BCAS 14d group. Thus, the progressive increase in resting Ca^2+^ in the astrocytes post-BCAS surgery may limit their ability to respond to external stimuli (i.e., low O_2_).

While low-O_2_-induced increases in astrocyte Ca^2+^ have been reported in various conditions such as stroke [47] and chemical ischemia [48], reductions in Ca^2+^ are less clear. One possible explanation for the reductions in Ca^2+^ in response to low O_2_ could be depletion or rundown in the slice preparation. If so, this would mean that only a small proportion of astrocytes from control mice could sustain the slicing procedure and respond to mild ischemia induced changes ex vivo. Our protocol induces mild ischemia (20% O_2_ for 5 min), which, when compared to other conditions such as oxygen–glucose deprivation (OGD) [49], may not be enough to trigger a Ca^2+^ response. Alternatively, and consistent with the findings of Fordsmann et al. 2019 [47], astrocyte activity in young mice is indeed reduced under acute hypoxic conditions, which may relate to different neuroprotective mechanisms between young and aged mice. In the Fordsmann et al. study (conducted in an animal model of stroke), reduced astrocyte Ca^2+^ activity in the electrically suppressed penumbra was observed in young mice but increased in older mice (18–24 months).

To date, no studies have monitored astrocyte Ca^2+^ dynamics in BCAS mice, limiting our understanding of the functional association between astrocyte Ca^2+^ events post-BCAS surgery and their inflammatory phenotype (pro- vs. anti-inflammatory). This study focused on a limited number of mRNA inflammatory markers corresponding to putative A2 and A1 phenotypes [50]; future research should include more pro- and anti-inflammatory markers to comprehensively assess the phenotypic changes of the astrocytes post-BCAS. Our data, however, would suggest that BCAS 14d and 28d mouse brains showed evidence of an anti-inflammatory or neuroprotective state as shown by a significant increase in the expression of S100A10 [51,52], IL-10 [53], Nrf2 [54], and TRPA1, all of which have been linked to ischemia-induced neuroprotection. However, a more extensive analysis of the expression of pro- and anti-inflammatory astrocyte markers with their reactive Ca^2+^ dynamics is needed to determine further if the observed functional changes predict the phenotype of glial cells.

TRPA1 ion channels are expressed in neurons and astrocytes [55–57]. These channels respond to cold temperatures and pain [49], and recent evidence suggests TRPA1 acts as a biosensor for O_2_ [46,58]. Because astrocytes can sense O_2_ [46], it is possible that under conditions of increased energy demand, such as chronic hypoperfusion to the brain, the activity of TRPA1 is increased. Kakae et al. (2023) reported exacerbated cognitive dysfunction and white matter damage in TRPA1 knockout mice following BCAS surgery. Notably, stimulation of TRPA1 ion channels with cinnamaldehyde ameliorated deleterious BCAS-associated outcomes [40]. Supporting a protective role, Pires et al. showed a neuroprotective effect of TRPA1 following stroke where TRPA1 channel activation caused vasodilation [59]. The pro-inflammatory role of TRPA1 channels may be linked to their inactivation by oxidative stress, which may depend on the degree of ischemia. In our study, the lack of functional changes to cortical neurons and the increased expression of anti-inflammatory markers would suggest mild ischemia-favored recovery-mediated pathways. Thus, TRPA1 channel regulation may provide new therapeutic targets for ischemic diseases, particularly in improving blood flow and reducing neuroinflammation.

### 4.3. Structural Changes in Microglia

In further support of a predominant pro-recovery state, we also show that at the structural level, cortical microglia showed dynamic changes characterized by an increased arborization at 14d post-BCAS surgery, followed by a decrease by day 28. The association between microglia structural, functional, and molecular changes is complex and influenced by the activity of neighboring astrocytes. Our study observed a greater than 3-fold increase in IL-10 in both BCAS 14d and 28d. Both astrocytes and microglia are responsive to IL-10, raising the possibility that the mild ischemia induced by the BCAS surgery, as proposed by Recasens et al. 2019, evokes the release of TGFβ, IL-2, and CXCL10, which, through IL-10, contributes to the microglia’s anti-inflammatory state [53].

### 4.4. Study Limitations and Future Research

In this study, we present evidence for progressive, dynamic changes to key elements of the NVU, namely, vascular cells, astrocytes, microglia, and neurons. At the vascular level, we observed impairments to hypoxia and adenosine-induced vasodilation. At the glial level, our data support the potential activation of anti-inflammatory or adaptive processes. Our study, however, poses a few limitations: (1) we did not evaluate if more severe chronic ischemia would shift putative neuroprotective mechanisms to a pro-inflammatory state, or if more severe conditions would further increase the magnitude of the Ca^2+^ events in astrocytes as observed in pathological conditions; (2) adaptive mechanisms to ischemia may be age-dependent, and our study was conducted in young mice; (3) our study was limited to a few glial-derived markers and we did not use additional structural analysis approaches (e.g., Sholl) to assess microglia arborization. Future studies in older mice and with animal models that evoke a more severe progressive ischemia (e.g., the ameroid constrictor model [60]) are warranted as these may better recapitulate the impact of progressive hypoperfusion, which is more prominent in the aging population.

## Data Availability

Data will be made available by the authors upon reasonable request.
